# Case Series: Managing Severe Mental Illness in Disaster Situation: the Croatian Experience After 2020 Earthquake

**DOI:** 10.3389/fpsyt.2021.795661

**Published:** 2022-02-02

**Authors:** Sara Medved, Azijada Srkalović Imširagić, Igor Salopek, Dragan Puljić, Hrvoje Handl, Marina Kovač, Alma Mihaljević Peleš, Danijela Štimac Grbic, Luka Romančuk, Roberto MuŽić, Laura Shields Zeeman, Martina Rojnić Kuzman

**Affiliations:** ^1^Department of Psychiatry and Psychological Medicine, Zagreb University Hospital Centre, Zagreb, Croatia; ^2^Neuropsychiatric Hospital “Dr Ivan Barbot”, Popovača, Croatia; ^3^Department of Psychiatry, General Hospital Karlovac, Karlovac, Croatia; ^4^University Psychiatric Clinic “Sveti Ivan”, Zagreb, Croatia; ^5^School of Medicine, University of Zagreb, Zagreb, Croatia; ^6^Croatian Institute of Public Health, Zagreb, Croatia; ^7^Department of Mental Health Prevention, Trimbos Institute (Netherlands Institute of Mental Health and Addiction), Utrecht, Netherlands

**Keywords:** severe mental illness, crisis, community mental health team, earthquake, CMHT

## Abstract

On the 29th of December 2020, amidst the COVID-19 pandemic, Petrinja in the Croatian Sisak-Moslavina County experienced a strong earthquake, resulting in a severe disruption in mental health service delivery. Specialized care community mental health teams were introduced days within the event with the aim to bridge the gap in psychiatric care that was severely disturbed in the region affected by the earthquake. Through a case series of patients with SMI, we describe how care was quickly deployed and delivered after a natural disaster and during a pandemic resulting in their functional recovery. Community mental health teams have the potential to provide feasible, comprehensive, and accessible mental health services, and their continued implementation in the post-disaster period in Croatia could be beneficial for care management of people with severe mental illness.

## Introduction

An earthquake of 6.4 moment magnitude (Mw) hit Petrinja in Croatian Sisak-Moslavina County on 29th of December 2020, which resulted in severe structural damage in the region and seven death causalities (1). It was also the second earthquake of that magnitude in Croatia that year; in March 2020, a 5.4 Mw earthquake hit the city of Zagreb, the capital of Croatia, but it did not affect Sisak-Moslavina County substantially (2). During the period when Petrinja earthquake hit, Croatia was facing a second lockdown to curb the number of COVID-19 cases. The health system and health personnel were overloaded with cases of COVID-19 nationwide (3).

Prior research shows that after exposure to severe trauma, such as an earthquake, the general population is at higher risk of developing psychiatric problems, especially post-traumatic stress disorder (PTSD), depression, and anxiety symptoms (4, 5). A significantly higher number of people show depressive and psychotic symptoms for months, even years after an earthquake has occurred (6–8). Furthermore, acute stress after these catastrophic events seldom increases the acute development of stress related disorders, such as peptic ulcer, hypertension, asthmatic attack, and ulcerative colitis (9–11). People with existing mental health issues, particularly severe mental illness (SMI), are even more susceptible to the adverse impacts of stressful events and to developing concomitant symptoms and mental health issues (12). People with SMI may also not have sufficient coping mechanisms or social resources to cope with these stressors or traumatic events (13). Also, due to a significant number of comorbidities and a shorter lifespan than general population (14, 15), people with SMI may be prone to the worsening of somatic conditions. Early intervention after trauma reduces acute stress and prevents psychological disturbances and, consequently, stress related somatic symptoms, including cardiovascular, gastric, pulmonary, etc. (16).

The majority of studies that focused on interventions in humanitarian crises have been conducted in low- and middle-income countries (17–19). In high-income countries, earthquakes in Kobe, L'Aquila, and Tohoku were the most investigated (20). Humanitarian research is often conducted in partnership with humanitarian organizations that are responding to the crisis (21). One of those organizations set standards to be applied in humanitarian response^1^, emphasizing the need for accessible health services to people with psychosocial disabilities^2^. Natural disasters are and will continue to represent a great challenge in addressing mental health issues globally (22, 23). Implementation of mental health policies that focus on prevention and improving crisis response in care delivery are important to support populations affected by natural disasters (24). Since the COVID-19 pandemic, several studies have described how double disasters (i.e., natural disasters coupled with the COVID-19 pandemic) affect the mental health of whole communities(25–30).

The scientific and clinical community have advocated for the development and implementation of community-based services for people with mental illness (23). Community mental health teams (CMHT) providing specialized community-based mental health care are one example of a service delivery model that can support care outcomes for people with mental illness, and many countries have integrated these teams as a part their overall mental health service infrastructure. CMHT can support people with mental illness in achieving their recovery goals in and around their community and provide appropriate specialized care in the event of a crisis to prevent unnecessary psychiatric-related hospitalization (31, 32).

This paper will present a case series study that consist of five case studies reporting on the deployment of CMHT in affected regions up to 7 months after the 2020 Petrinja earthquake to provide insights on the method and care delivered to manage the mental health of people with SMI. The cases will demonstrate the possibility of CMHTs to early detect, monitor, and manage SMI in disaster situations.

## Case Descriptions

Prior to the earthquake in 2020, mental health care in the Sisak-Moslavina County was provided by both the Dr. Ivan Barbot neuropsychiatric hospital and the Dr. Ivo Pedišić general hospital serving a population of ~160,000 inhabitants. After the earthquake, the psychiatric department within the “Dr. Ivo Pedišić” general hospital was repurposed for non-psychiatric use. The earthquake and COVID-19 pandemic resulted in only emergency services being maintained (33, 34). Emergency medical service (EMS) staff is usually not sufficiently trained in mental health care (35). Public transportation within the County was reduced and, in some places, stopped entirely, many private cars were damaged. As a result, regular psychiatric outpatient care in the County was hardly accessible for the majority of the population. Croatia has several years of experience in the implementation of CMHTs, partially through a European Commission project LaRge-scalE implementation of COmmunity based mental health care for people with seVere and Enduring mental ill health in EuRopE (RECOVER-E) (31) which focuses on the development, implementation, and evaluation of CMHTs for SMI in five sites in five Central and Eastern European countries. Following the joint initiative of the Croatian Psychiatric Association (CPA), RECOVER-E project experts from Croatia and the Dr. Ivan Barbot hospital, the Ministry of Health endorsed the establishment and deployment of CMHTs on January 3rd, 2021 for Sisak-Moslavina County. The Croatian Institute of Public Health provided the support to CMHTs from May 2021 until August 2021 when all previously existing psychiatric services have returned in function and the need for CMHTs diminished. CMHTs were funded by the Croatian Health Insurance Fund within the compulsory health insurance package. The structure of CMHT members was adopted from RECOVER-E project and adjusted to reflect local needs and opportunities (31). Members of the CMHT included at least one psychiatrist, psychiatry resident, psychiatric nurse, psychologist, and in some instances a child and adolescent psychiatrist. CMHTs included local mental health professionals from Dr. Ivan Barbot hospital and professional volunteers from the CPA. CMHTs provided on-site crisis resolution, psychological interventions, psychopharmacological home treatment, remote consultations, and a range of flexible interventions depending on specific needs such as liaising with the local general hospitals in case of emergent somatic comorbidity, liaising with general practitioners (GP) where medication prescription was needed, and liaising with other rescue teams in case of severe home damage related to the earthquake. The teams were situated in three major centers of the County, Petrinja, Sisak, and Glina, where temporary psychiatric ambulances separate from EMS were established. The information about implementation was shared through the local and national media, and informative brochures with contacts for CMHTs were distributed to first responder organizations, local civil defense, non-governmental organizations (NGO), and community health centers. The intervention was available to those who expressed the need for psychiatric care and to those who were detected in need by the community. A flexible team structure enabled all age groups to be treated. The patients would be followed by the CMHT until returning to their previous level of functioning or declining further visits. The rate of declining was not investigated; however, there were obviously no contraindications since the team did not oppose the any discontinuation. From January 4th 2021 to August 1st 2021, CMHTs provided altogether 758 interventions to the people in Sisak-Moslavina County. Most interventions were provided to people suffering from stress related disorders (81%), and 9.3% for people with SMI. Of all interventions, 3.6% were provided for non-psychiatric reasons, such as gastritis, hypertension, and arrythmias. Other interventions (6.1%) included support for people with dementia, alcohol related disorders, insomnia, intellectual disabilities, behavioral and emotional disorders in childhood, and personality disorders. Five cases of patients demonstrate the role of CMHT in early detection, monitoring, and management of SMI.

### Case 1

Seven days after the earthquake, a local NGO informed the CMHT about a patient in a possible acute mental health crisis in a remote, hard to reach community. The team managed to visit the patient within 2 days. A 57-year-old female appeared unwilling to participate in the intersection with the CMHT, appeared tense, depressed, and frightened. She was living with her son who described those symptoms have been present for more than a month. He informed their GP about her condition, but due to the disruption in healthcare services, they did not receive follow-up support or care. During the conversation, she would occasionally stare at one point on the celling but denied having any hallucinations. She confirmed that she was having difficulty sleeping and experienced anxiety. She accepted treatment support for the sleep troubles and anxiety and agreed to then have her mental health status re-assessed by the CMHT. The team prescribed her olanzapine 5 mg/daily. Three days after the first visit, her son contacted the team saying the patient refused to eat and drink. The CMHT requested the Croatian mountain rescue service to transport the CMHT to the patient, since the terrain was inaccessible for an EMS vehicle. Upon arrival, the patient refused to talk, and the son confirmed she muttered what seemed to resemble suicidal ideation. The team assessed this as a high-risk situation and took the patient and informed EMS about the arrival of the patient at the Dr. Ivan Barbot psychiatric hospital for inpatient admission. She was later diagnosed with unspecified psychosis and discharged after 3 weeks of hospitalization in an improved condition, however with negative symptoms still mildly present. By the end of July 2021, she continued to receive routine outpatient care at the Dr. Ivan Barbot psychiatric hospital.

### Case 2

Following a telephone call 2 weeks after the earthquake, the CMHT visited a patient in a dislocated municipality. The patient was already receiving mental health care for unspecified psychosis and complex PTSD since 2005 but was not able to visit a psychiatrist due to the lack of public transportation after the earthquake. He shared the household with one of his parents, was unemployed, and received social allowances as his only source of income. He was prescribed diazepam by a GP in doses from 30 to 60 mg per day as needed. However, the patient complained that diazepam was not helping anymore: he had parasuicidal thoughts and could not sleep for longer than an hour every night. He did not want to be admitted to the hospital but was willing to take medications. The team administrated olanzapine 5 mg and started immediately providing short supportive psychotherapy session. After the initial 45-min session, the patient reported slight improvement, did not report any suicidal thoughts and intentions, and he made a suicide prevention contract with the team. For coping with depressive symptoms, the patient was prescribed with fluvoxamine 100 mg/daily and olanzapine 10 mg/daily. He and his parent were provided with all necessary mental health service contacts. CMHT visited next week and provided the second supportive psychotherapy session. The patient described vanishing of all suicidal thoughts and achieving a good sleep routine. The team contacted his GP for further prescription of medication and performed weekly telephone sessions with the patient. During the last visit in June 2021, the patient reported being stable. He found few new activities, was taking care of abandoned animals, and started to engage into physical activity. By that time, public transport returned to operation, and he continued his ongoing care with his treating psychiatrist.

### Case 3

Approximately 2 weeks after the earthquake, the team was informed about a 44-year-old unemployed patient near Glina that was in psychiatric treatment since 2017 due to schizoaffective disorder and generalized anxiety disorder. His last psychiatric appointment was 6 months before the earthquake, when the psychiatrist continued with mirtazapine 30 mg/daily, paroxetine 20/daily, and pregabalin 450 mg/daily, and switched him from olanzapine 20 mg/daily to risperidone 4 mg/daily due to drowsiness. A few months before the earthquake, he was engaged in neurological treatment for restless leg syndrome and prescribed with ropinirole 8 mg/daily and diazepam 30 mg/daily. However, since the COVID-19 restrictions came into force and health services were disrupted after the earthquake, he was not able to stay in regular neurological outpatient visits. During the teams' first visit, the patient was anxious, seemed traumatized, had trouble sleeping, and prominent restless leg syndrome symptoms. He reported that he had severe limitations in functioning and felt unsafe in his home, despite the building being categorized safe to remain in by civil engineers employed by the municipality. The team re-prescribed olanzapine (10 mg/daily) and advised him to discontinue mirtazapine as a second antidepressant due to expected olanzapine induced drowsiness. The team provided supportive psychotherapy together with psychological debriefing. In the following week, the team gradually reduced doses of diazepam (to 20 mg/daily) and continued to provide supportive psychotherapy. The patient was followed until May 2021 in which anxiety and sleeping disturbances were reduced. He became more active and became a municipal maintenance worker. His restless leg syndrome improved, and the team gave him directions on continuing his neurological outpatient visits.

### Case 4

In the middle of January 2021, the CMHT was consulted to see a mistrustful 59-year-old woman in Petrinja diagnosed with schizophrenia and multiple somatic comorbidities. The team was contacted by her daughter reporting the patient being suspicious and refusing to take her long-acting injectable antipsychotic that she had last received a few weeks before the earthquake. There was also a lack of information about when the next injection would be received, as there was no communication with her treating psychiatrist. The patient refused to be seen by an EMS team but agreed to meet with members of the CMHT in Petrinja's community health center. During the visit, the patient confided to the team members about her paranoid beliefs. She reported that household members were attempting to poison her, and that she does not like to take long-acting injectable antipsychotics as she felt she no longer had schizophrenia. She also complained about pelvic discomfort and vaginal bleeding. Because of the high risk of further illness progression due to medication non-adherence and the lack of support system, all possibly resulting in deterioration of psychic and somatic health, the team indicated a further diagnostic evaluation and treatment, so the patient was transferred to Dr. Ivan Barbot hospital. During the 1-month in hospital stay, she was seen by a psychiatrist, internal medicine specialist, and a gynecologist, and diagnosed with residual schizophrenia, bacterial pneumonia, and cervical polyps. She was prescribed with haloperidol 7 mg/daily, promazine 25 mg/daily, and biperiden 2 mg/daily upon which her psychotic symptoms diminished. She was successfully treated for pneumonia and after discharge continued the gynecological monitoring in an outpatient clinic. Until the beginning of August 2021, she continued to experience stability in her psychiatric state.

### Case 5

After the earthquake, all beneficiaries from Counties' retirement homes were temporarily relocated to a hospice in the town of Topusko. In July 2021, 6 months after the earthquake, during the CMHT regular round of home visits, the team was notified about a beneficiary with previously diagnosed schizophrenia that started being hostile toward the hospice workers. The 47-year-old patient articulated that her body is not hers, and her sense of touch is unusual. During the placement in the Topusko hospice, she was treated for pulmonary tuberculosis, asthma, and COVID-19 infection with the introduction of quadruple antituberculosis therapy. She received regular mental health treatment, including 75 mg clozapine and 1.5 mg alprazolam daily. CMHT provided her with psychosocial support in the form of counseling and increased clozapine dose for 12.5 mg daily. One week after the initial visit, CMHT noticed an additional deterioration of her mental state in the form of paranoia toward other beneficiaries. Given the new circumstances, and despite the increased clozapine dosage, the possibility of antitubercular therapy-induced psychosis was considered. Therefore, CMHT transferred her to Dr. Ivan Barbot hospital where eventually the diagnosis of iatrogenic genesis of psychotic exacerbation was confirmed. Upon achieving the remission of psychotic symptoms, she was displaced to the Special Hospital for Pulmonary Diseases and Tuberculosis where she continued treatment for pulmonary tuberculosis.

## Discussion

This case series presents diverging experiences of mental health issues during the pandemic and in the aftermath of the 2020 earthquake. All cases demonstrate the importance of early detection of symptoms or changes in functioning and regular contact with patients to inform changes in mental health status or in circumstances.

We believe introducing CMHTs as a standard post-disaster mental health intervention could have several benefits. First, they improve access to psychiatric services which is often disrupted in disaster situation, such as in our experience. It is well-known that low levels of accessibility of mental health services increase the inequality in the provision of adequate care for people with SMI (36). The mental health services in general became less accessible during the pandemic in the majority of countries (33, 34). It was also noted that SMI patients had a higher level of morbidity and mortality due to COVID-19, and one of the explanations lies in the inadequate accessibility and provision of mental health services for SMI patients (37). Novel approaches to delivery of care during the pandemic have helped offset the psychiatric risks to SMI patients, and it is presumable that those approaches may be applicable to other disaster scenarios (12). The CMHT may represent the only accessible service in the event of an acute mental health crisis or given measures imposed during the COVID-19 pandemic.

Second, CMHTs can provide fast response to prevent further escalation of an acute mental health crisis. Early detection of symptoms has a positive impact on the outcome and recovery of the disease (38). Our CMHT was able to visit the patient in case number 1 within 48 h and used other available facilities in order to relieve the burden on EMS services. Implementation of mental health outreach teams immediately after severe earthquakes showed significant mental health benefits in Armenia, China, and Japan (39–43). After the 1988 Armenian earthquake, a psychiatric outreach program with trauma/grief-focused brief psychotherapy to adolescents was implemented, which was associated with a reduction of risk of developing PTSD and prevented the exacerbation of depressive symptoms (40, 41). In 2008, Sichuan earthquake psychological rescue services were quickly deployed as well as hotlines for psychological support have been implemented for people near the disaster areas, and also for general public (42). Brief mental health intervention provided demonstrated that overall patient-level results were extremely satisfactory, with marked improvements of functionality and/or symptom intensity seen in the affected population (43). After the 2011 Tohoku earthquake and tsunami, psychiatric mobile teams were immediately dispatched to work in collaboration with local mental health professionals (39). The teams supported them in continuing pre-disaster psychiatric services, providing on-site treatment of acute stress and psychoeducation at communal shelters (39). For adequate post-disaster service, planning, preparation, and management are essential (44). Timely interventions can be observed in countries with predetermined guidelines for post-disaster period (25, 39, 42). Croatia has several recommendations for psychosocial support in disaster situations (45, 46). However, the precise establishment and provision of outreach psychiatric care in crisis situation is not defined (45, 46).

Finally, the flexibility in formation of CMHTs makes them efficient in detecting a wide range of clinical situations. In these cases, the connection of CMHTs with GPs, EMS, and hospital allowed the resolution of highly complex somatic conditions, like detecting iatrogenic psychosis. Unfortunately, CMHTs are not commonly seen in South Eastern European countries, but there are several initiatives and pilot programs that aim to develop and sustain community-based mental health services, including CMHTs (31, 47, 48). They are intended mostly for patients with SMI as an enforcement for rehospitalization prevention and overall admission reduction. CMHT can be adjusted across different clinical groups, such as for persons with first episode psychosis, those with intellectual disabilities, forensic patients, etc., and the members can be formed of different specialists (e.g., some teams include employment specialists, police officers, or social workers) (49–51).

This paper as a case series report has several limitations. The primary limitation is lack of a comparison group (52). However, this limitation is inherent to case studies. The benefits of receiving outreach community psychiatric care for patients with SMI have been emphasized (31, 32), and we believe we provided SMI patients with above-standard care especially in the situation where standard care is not available at all. CMHTs after 2020 Petrinja earthquake were conducted on the back of numerous highly devoted professional volunteers. Those professionals do not necessary reflect the overall mental health workforce in Croatia. Selection bias might also be present, since only patients who agreed to receiving treatment by the CMHT were included in this particular case series. However, we believe this case series provides valuable information about the management of patients with SMI in double disaster using CMHTs and will lead to more extensive research in the field of outreach post-disaster services.

In sum, after a disaster, CMHTs have the potential to deliver feasible, comprehensive, and accessible mental health services in remote areas affected by a disaster or impacted more substantially due to public health measures in the place, for instance, in the event of the COVID-19 pandemic.

## Data Availability Statement

The original contributions presented in the study are included in the article/supplementary files, further inquiries can be directed to the corresponding author/s.

## Ethics Statement

Ethical review and approval was not required for the study on human participants in accordance with the local legislation and institutional requirements. The patients/participants provided their written informed consent to participate in this study. Written informed consent was obtained from the individual(s) for the publication of any potentially identifiable images or data included in this article. All patients in this case series gave informed consent for the publication.

## Author Contributions

SM, AI, IS, DP, HH, RM, LR, MK, AP, DŠ, and MRK participated in the project and in the study. SM, AI, IS, and DP participated in the writing of the first draft of the manuscript, gave critically relevant comments in the revised version of the manuscript study, and approved the final version of it. HH, RM, and LR gave critically relevant comments and approved the final version. MK, AP, LZ, and DŠ gave critically relevant comments, participated in the writing of the final revision of the manuscript, and approved the final version of it. MRK revised the first draft of the study, and gave critically relevant comments on the subsequent revisions of the manuscript, and approved the final version of it. All authors contributed to the article and approved the submitted version.

## Funding

The implementation of the psychiatric mobile team service was funded by the Croatian Health Insurance Fund according to their diagnostic and therapeutic procedures codebook as a part of compulsory health insurance in Croatia. The Croatian Institute of Public Health initiated and funded the project Preservation of mental health in the earthquake-affected area in the Sisak-Moslavina County in the period of May 15^th^ to August 15^th^ 2021 within which SM, IS, HH, and RM received funds for assistance during the service delivery in the County.

## Conflict of Interest

The authors declare that the research was conducted in the absence of any commercial or financial relationships that could be construed as a potential conflict of interest.

## Publisher's Note

All claims expressed in this article are solely those of the authors and do not necessarily represent those of their affiliated organizations, or those of the publisher, the editors and the reviewers. Any product that may be evaluated in this article, or claim that may be made by its manufacturer, is not guaranteed or endorsed by the publisher.

## Footnotes

^1^Sphere Association. About (2018). Available online at: https://spherestandards.org/about/ (accessed December 1, 2021).

^2^Sphere Association. The Sphere Handbook Humanitarian Charter and Minimum Standards in Humanitarian Response (2018). Available online at: https://handbook.spherestandards.org/en (accessed December 1, 2021).
