# Supplementary material for: Case Series: Managing Severe Mental Illness in Disaster Situation: the Croatian Experience After 2020 Earthquake
Source: Front Psychiatry. 2022 Feb 2;12:795661. doi: 10.3389/fpsyt.2021.795661 (PMC8847377; doi:10.3389/fpsyt.2021.795661)
Supplement: Supplementary file 1 [file Data_Sheet_1.PDF]

CARE
case report guidelines

CARE Checklist of information to include when writing a case report

(cc) BY-NC-ND

Topic	Item	Checklist item description	Reported on Line
**Title**	1	The diagnosis or intervention of primary focus followed by the words "case report"	Page 1, line 1
**Key Words**	2	2 to 5 key words that identify diagnoses or interventions in this case report, including "case report"	Page 1, line 18
**Abstract (no references)**	3a	Introduction: What is unique about this case and what does it add to the scientific literature?	Page 1, line 20
	3b	Main symptoms and/or important clinical findings	
	3c	The main diagnoses, therapeutic interventions, and outcomes	Page 1, line 24
	3d	Conclusion—What is the main "take-away" lesson(s) from this case?	Page 1, line 26
**Introduction**	4	One or two paragraphs summarizing why this case is unique (**may include references**)	Page 1, line 31
**Patient Information**	5a	De-identified patient specific information.	Page 4, line 122
	5b	Primary concerns and symptoms of the patient.	Page 4, line 125
	5c	Medical, family, and psycho-social history including relevant genetic information	
	5d	Relevant past interventions with outcomes	Page 4, line 126
**Clinical Findings**	6	Describe significant physical examination (PE) and important clinical findings.	Page 4, line 128
**Timeline**	7	Historical and current information from this episode of care organized as a timeline	Page 4, line 122
**Diagnostic Assessment**	8a	Diagnostic testing (such as PE, laboratory testing, imaging, surveys).	
	8b	Diagnostic challenges (such as access to testing, financial, or cultural)	Page 4, line 128
	8c	Diagnosis (including other diagnoses considered)	
	8d	Prognosis (such as staging in oncology) where applicable	
**Therapeutic Intervention**	9a	Types of therapeutic intervention (such as pharmacologic, surgical, preventive, self-care)	Page 4, line 131
	9b	Administration of therapeutic intervention (such as dosage, strength, duration)	Page 4, line 131
	9c	Changes in therapeutic intervention (with rationale)	Page 4, line 135
**Follow-up and Outcomes**	10a	Clinician and patient-assessed outcomes (if available)	
	10b	Important follow-up diagnostic and other test results	Page 4, line 139
	10c	Intervention adherence and tolerability (How was this assessed?)	Page 3, line 112
	10d	Adverse and unanticipated events	
**Discussion**	11a	A scientific discussion of the strengths AND limitations associated with this case report	Page 7, line 269
	11b	Discussion of the relevant medical literature **with references**.	Page 6, line 222
	11c	The scientific rationale for any conclusions (including assessment of possible causes)	Page 6, line 227
	11d	The primary "take-away" lessons of this case report (without references) in a one paragraph conclusion	Page 7, line 280
**Patient Perspective**	12	The patient should share their perspective in one to two paragraphs on the treatment(s) they received	Page 7, line 284
**Informed Consent**	13	Did the patient give informed consent? Please provide if requested	Yes ☒ No ☐
